# Flower opening dynamics, pollen-ovule ratio, stigma receptivity and stigmatic pollen germination (in-vivo) in *Chaenomeles speciosa* (Sweet) Nakai

**DOI:** 10.1038/s41598-024-57655-1

**Published:** 2024-03-26

**Authors:** Xianqin Wan, Dongchan Sun, Chao Gao

**Affiliations:** Institute for Forest Resources and Environment of Guizhou, Key Laboratory of Forest Cultivation in Plateau Mountain of Guizhou Province, College of Forestry, Guizhou University, Jiaxiu South Road, Guiyang, 550025 China

**Keywords:** *Chaenomeles speciosa*, Flowering phenology, Breeding system, Pollen, Plant sciences, Plant breeding, Plant reproduction

## Abstract

Although *Chaenomeles* is widely used in horticulture, traditional Chinese medicine and landscape greening, insufficient research has hindered its breeding and seed selection. This study investigated the floral phenology, floral organ characteristics, palynology, and breeding systems of *Chaenomeles speciosa* (Sweet) Nakai. The floral characteristics of *C. speciosa* were observed both visually and stereoscopically. The microstructures of the flower organs were observed using scanning electron microscopy. Pollen stainability was determined using triphenyl tetrazolium chloride staining. Stigma receptivity was determined using the benzidine-H_2_O_2_ method and the post-artificial pollination pollen germination method. The breeding system was assessed based on the outcrossing index and pollen–ovule ratio. The flowers of *C. speciosa* were bisexual with a flowering period from March to April. The flowering periods of single flowers ranged from 8 to 19 d, and those of single plants lasted 18–20 d. The anthers were cylindrical, with the base attached to the filament, and were split longitudinally to release pollen. The flower had five styles, with a connate base. The ovaries had five carpels and five compartments. The inverted ovules were arranged in two rows on the placental axis. The stigma of *C. speciosa* was dry and had many papillary protrusions. In the early flowering stage (1–2 d of flowering), the pollen exhibited high stainability (up to 84.24%), but all stainability was lost at 7 d of flowering. Storage at – 20 °C effectively delayed pollen inactivation. The stigma receptivity of *C. speciosa* lasted for approximately 7 days, and the breeding system was classified as outcrossing with partial self-compatibility.

## Introduction

*Chaenomeles* originated in China, with more than 700 cultivars found in nurseries^1^. To date, there are approximately 22 wild and 21 cultivated species of *Chaenomeles* worldwide, including approximately 14 wild and 7 cultivated species in China^2^. *Chaenomeles,* which has a long history of cultivation in China^3^ and profound cultural heritage, is characterized by strong ornamental value, rich variety of resources, strong adaptability to different soils and environments, and excellent traits. Because of these characteristics, *Chaenomeles* has always played an important role in landscape design and ecological environment construction practices^4^. Furthermore, *Chaenomeles* has high nutritional value, and its flowers, fruits, and leaves contain various nutrients, such as vitamins, organic acids, flavonoids, and sugars^5–7^. Additionally, *Chaenomeles* has high research value as a pollinating tree^8,9^ and as a grafting rootstock^10^ for apple trees; these plants can significantly improve the quality of apple fruits; therefore, *Chaenomeles* is highly important for the cultivation and seed selection of new apple cultivars. In China, however, the breeding of *Chaenomeles* started relatively late and mainly depends on hybrid breeding and selective breeding, and the main ornamental cultivars were introduced from North America, Europe, and other places. Furthermore, its application is limited by various factors, such as cultivar scarcity and an overemphasis on spring flower landscaping. In addition, current research on *Chaenomeles* mainly focuses on its fruits and leaves rather than its flowers. Since 2002, intensive selective breeding of new *Chaenomeles* cultivars has been practiced in China^11,12^, and some research has been conducted on the mechanisms underlying flower color changes^13^ and leaf color changes^14^.

*Chaenomeles speciosa* (Sweet) Nakai, distributed in central, eastern, and southwestern China, along with *C. cathayensis*, *Malus micromalus*, and *Malus halliana,* are collectively known as the “Four Cultivars of *Chaenomeles*”. *C. speciosa* is currently cultivated worldwide^15^ and has the characteristics of drought resistance, cold resistance, and barren soil tolerance in addition to light preference and high sensitivity to dampness and waterlogging. It is an excellent garden tree species for spring flower display and autumn fruit production. In recent years, *C. speciosa* has been widely used in courtyard and road greening and potted landscape production, and screening excellent *C. speciosa* varieties has become one of the important goals of breeding selection. To date, however, most of the studies on *C. speciosa* have focused on its dual use as a Chinese herbal medicine and functional food^16,17^. The chemical composition of the extract of its fruit is rich in flavonoids, phenols, terpenes, and phenylpropanoic acid^18,19^, which have antitumor^20^, anti-inflammatory^21^, antioxidant^22^, analgesic^23^, and antibacterial^24^ functions. However, studies on its flowering biology and breeding system are scarce and superficial, and the related research has been limited to its floral morphological characteristics, pollination habits, and pollen storage characteristics^25,26^. These conditions strongly affect the further popularization of *C. speciosa*.

The flowering biology and breeding system of plants constitute the foundation and main focus of plant reproductive research. Systematic research in these respects can provide a deep understanding of the reproductive characteristics of flowering plants as well as a theoretical basis for the management and regulation of the flowering period of landscape plants, the selection of new cultivars, and the promotion of their application in gardens. The flowering biology of plants mainly includes floral composition, flowering patterns, flowering phenology, flower display, pollination, development and aging^27^. Different floral compositions and flower opening styles have an impact on the breeding system of plants. In turn, the breeding system of plants shows a certain degree of adaptability to the development of internal organs and external morphological characteristics of flowers^28^. The breeding system of plants mainly consists of the comprehensive characteristics of flowers, the lifespan of various sexual floral organs, the degree of self-compatibility and the mating system, with the mating system being the central part^29^. Because the mating system can have a major impact on changes in plant morphological characteristics and evolutionary direction, it has become a popular topic of research in evolutionary biology^30^. The outcrossing index^31^, pollen‒ovule ratio^32^, and bagging pollination are important methods for investigating different plant breeding systems^33^. The flowering phenology of plants can be quantified based on indices such as the initial flowering date, the final flowering date, the number of flowers and the floral synchronicity index^34^. Flowering phenology reflects the influence of genetic and environmental factors on the flowering patterns of plants and is used mainly for research on plant flowering patterns, the effect of abiotic factors, the genetic basis of the flowering plant, its natural selection and its adaptive significance, thereby constituting one of the important characteristics of plants. The manner in which flowers are displayed is crucial for attracting pollinators^35^, and in addition, the diversity of plant breeding systems is often reflected in the floral and pollination characteristics of plants^28^.

Based on the aforementioned findings, we investigated the characteristics of the flowering phenology and floral organs of *C. speciosa* and evaluated its breeding system based on observations. The findings of this study might provide a theoretical foundation for research on the reproductive biology of *C. speciosa*, its florescence management and new cultivar breeding.

## Results

### Opening dynamics of single plants

**Figure 1:**
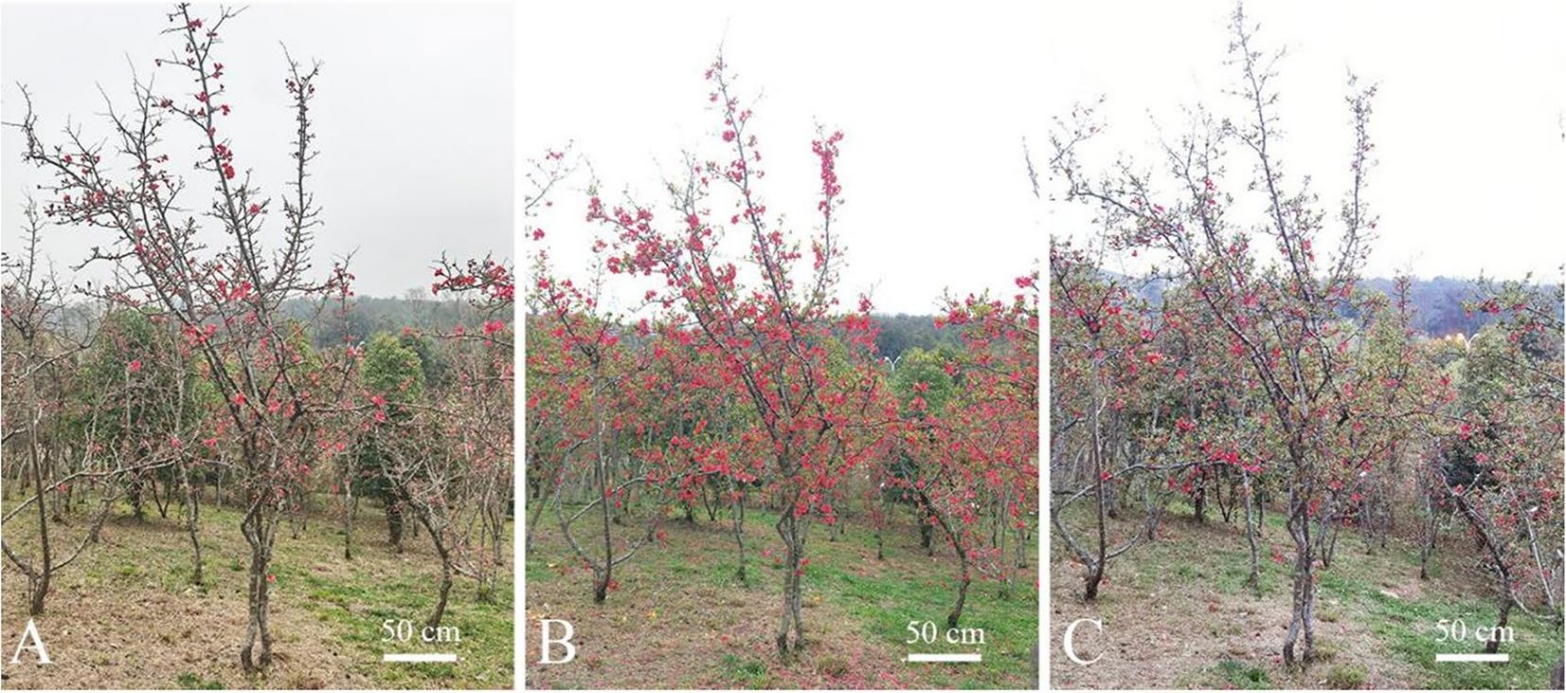
Opening dynamics of single *Chaenomeles speciosa* plants. (**A**) Early flowering stage. (**B**) Blooming stage. (**C**) Late flowering state.

The inflorescence of *C. speciosa* was cymose and composed of 3–5 small umbellules with 2–3 flower clusters, and the total number of flowers in the entire inflorescence was 8–15. In the same inflorescence, the small umbellule at the top opened first, followed by the surrounding umbellules. In each umbellule, the flower on the top opened first. The *C. speciosa* plants underwent nutritional growth, and after a period, the buds began to differentiate and entered reproductive growth. The opening dynamics of single *C. speciosa* plants lasted 18–20 d in a concentrated flowering manner. The flowering period in the investigated area was from March to April. At the beginning of March, the plants entered the early flowering stage (Fig. 1A), which lasted for 4–5 d. During this stage, the top crowns of most flower buds began to loosen and were about to open. Subsequently, the blooming stage (Fig. 1B) lasted for 12–15 d. In this stage, the flower buds were almost fully opened, and the vitality of each flower organ reached the most vigorous level; this was also the best period for viewing. In late March, most of the petals fell, and the plants entered the late flowering stage (Fig. 1C), which lasted for 3–5 d. Although the calyx segments did not show signs of withering, the stigma became smaller and darker due to withering.

### Opening dynamics of single flowers

The opening processes of single flowers of different *C. speciosa* plants were basically the same, lasting for 8–10 d. Flowers opened before or synchronously with the leaves. The end of the single-flower lifespan was marked by the loss of pollen stainability, complete shedding of petals, and loss of stigma receptivity. The anthers dispersed a large amount of pollen within 1–2 d of the flowering period, with partial or no pollen dispersion occurring at low temperatures on rainy days. The single flowers were divided into four stages according to their morphological characteristics.

**Figure 2:**
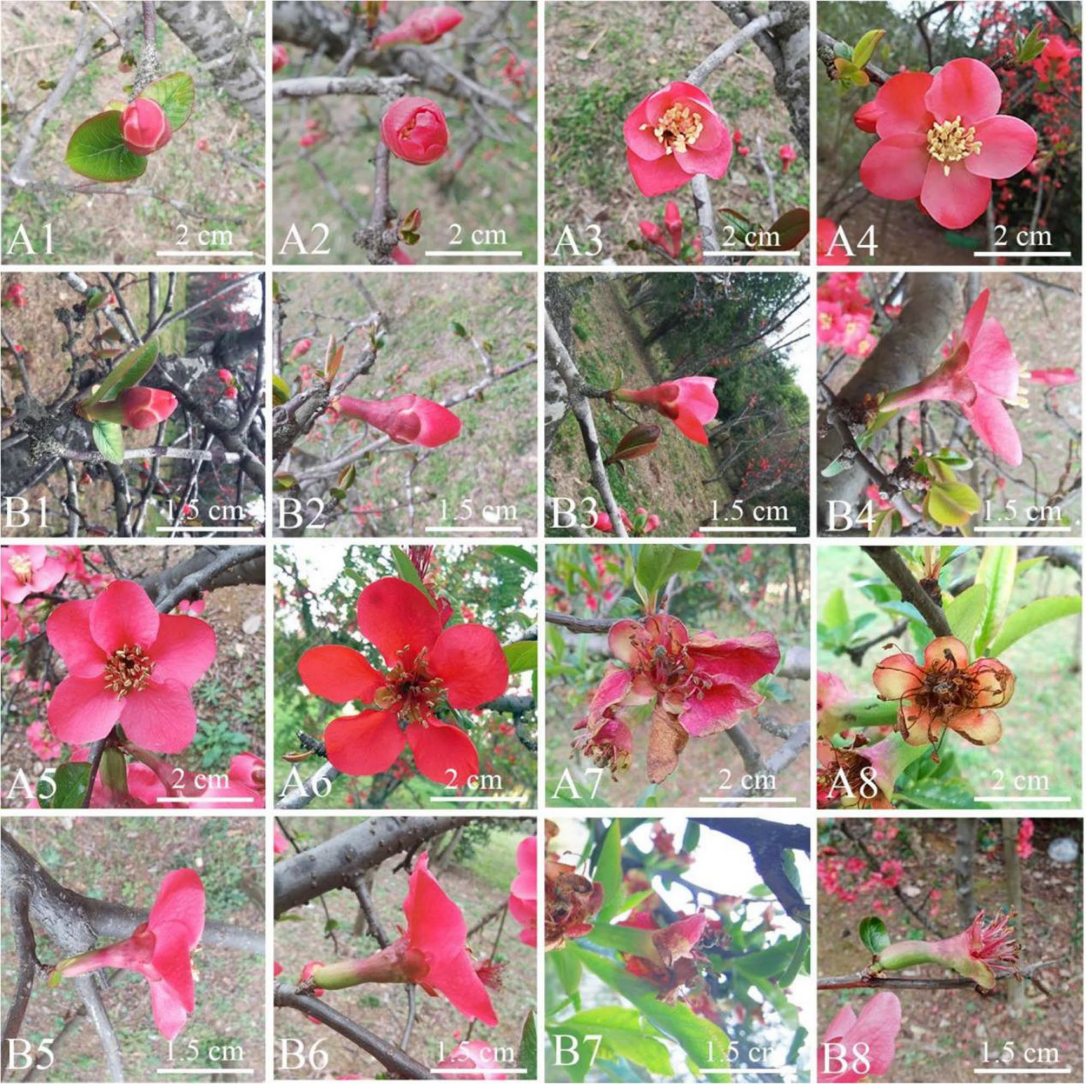
Opening dynamics of single *Chaenomeles speciosa* flowers. (**A1**) Front view of a single flower in the bud break stage. (**B1**) Lateral view in the bud break stage. (**A2**,**A3**) Front view in the flowering stage. (**B2**,**B3**) Lateral view in the flowering stage. (**A4**–**A6**) Front view in the blooming stage. (**B4**–**B6**) Lateral view in the blooming stage. (**A7**,**A8**) Front view in the withering stage. (**B7**,**B8**), Lateral view in the withering stage.

1. Bud break stage (Fig. 2A1,B1). The buds gradually expanded, and the bracts began to loosen. The petals wrapped tightly around the pistils and stamens. The red part of the petals was exposed, and the anthers appeared slightly yellow. The stigma was light pink with two converging cracks. Afterward, the buds continued to grow, and the petals gradually loosened. This stage lasted for 2–3 d.
2. Flowering stage (Fig. 2A2,A3,B2,B3). The flowers of *C. speciosa* began to open. The pistils and stamens were lower than the petals. The stigma appeared light red, the anthers were bright yellow, and the filaments were white. This stage lasted for 1–2 d, at which point no pollen dispersion was observed.
3. The blooming stage (Fig. 2A4–A6,B4–B6). The corolla completely opened and was radially symmetrical. With the growth of the flower, the diameter of the flower reached its maximum, the petal gradually changed from red to crimson, and the stamen cluster expanded radially. The stamens and pistils were higher than the petals; the stigma gradually turned bright yellow, extended vertically and gained receptivity; and the filaments gradually turned light red. The anthers shrank, and a large amount of pollen was released. At 1 d after flowering, pollen release began, followed by a peak period of release. This stage lasted for 4–5 d.
4. The withering stage (Fig. 2A7,A8,B7,B8). The petals shrank noticeably, and the color changed from crimson to brown. As the flowers gradually withered, the petals fell off, the stigma withered (the color changed from bright yellow to black‒brown), the anthers withered (the color changed from yellow to black‒brown), the pollen lost stainability, and the filaments became crimson. This stage lasted for 3–5 days.

### Characteristics of floral organs

**Figure 3:**
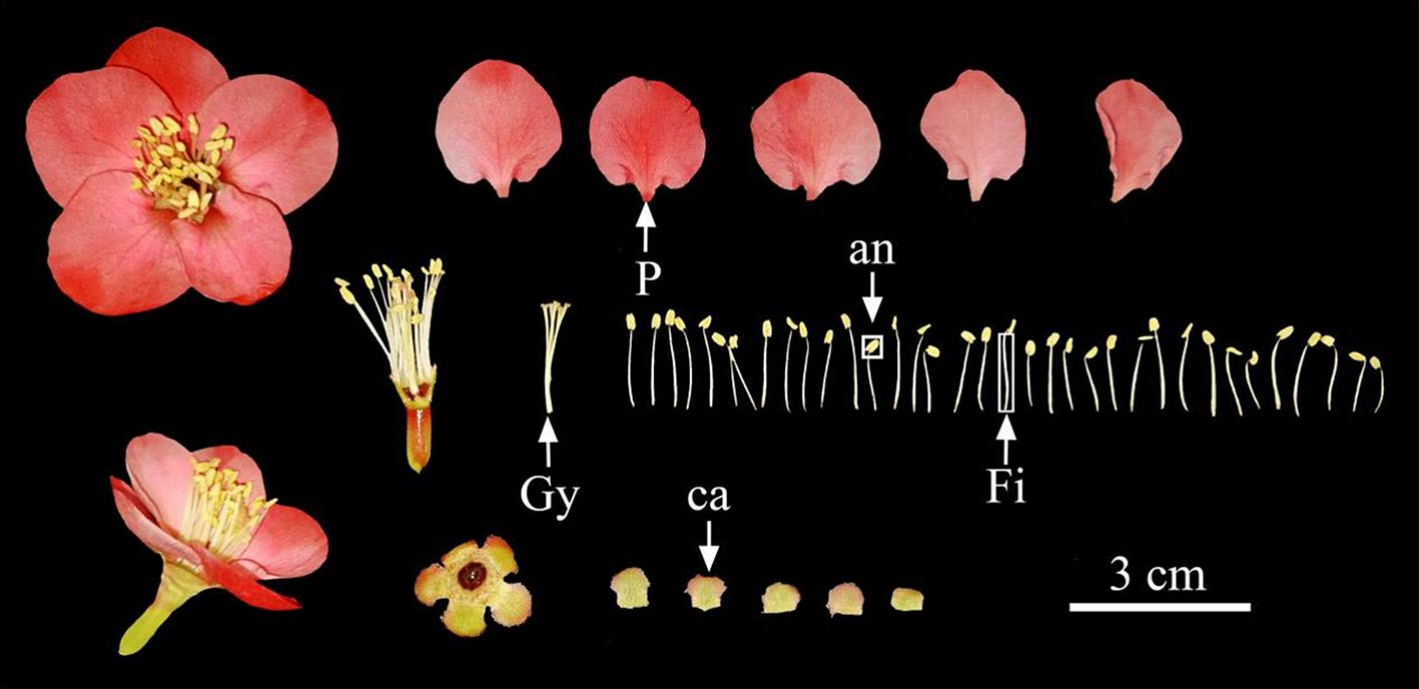
Characteristics of the floral organs of *Chaenomeles speciosa.* an, anther; ca, calyx; Gy, gynoecium; P, petal; Fi, filament.

Observations of the floral organs of *C. speciosa* showed that the flowers are bisexual, with a flowering duration of 3–4 months. Three to five flowers were clustered on old biennial branches. The flowers were fragrant with a diameter of 3–5 cm. The corolla showed a rose-shaped radial symmetry, with the petals and sepals arranged in an overlapping, tiled pattern within the bud. The pedicel was short and thick, approximately 3 mm long or nearly sessile. The calyx tube was prominent, with the central part sunken downward. It fused with the lower part of the perianth and filaments, forming a barrel-like shape. The tube had no hair on the outside. The flowers had five sepals, which were 6.78 ± 0.38 mm long and 7.05 ± 0.27 mm wide and had a crimson color. The sepals had a rounded and blunt apex, with flat or wavy edges and yellowish-brown eyelashes. The flowers had five petals, which were 20.30 ± 1.26 cm long and 18.42–1.18 mm wide and had an obovate or round shape. The base of the petal extended into a short claw, which was 10–15 mm long and 8–13 mm wide with a scarlet and a light red or white color. The pistils were slightly taller than or flush with the stamens. The stamens grew separately, and the number of stamens was approximately 30–35. The filaments were separated from each other and were 15.98 ± 1.97 mm long and 0.72 ± 0.12 mm wide. In the early stage of growth, the filaments were white and became scarlet in the late stage. There were five styles with a converged base, with a length of 16.20 ± 0.46 mm and width of 1.29 ± 0.04 mm. The plants had no hair or were slightly hairy. The stigma had a head-like shape with indistinct divisions and was equivalently long to the stamen. The base of the anther was attached to the top of the filament, and the anthers were basifixed. After maturity, the anther split longitudinally along the long axis to disperse pollen. The characteristics of the floral organs of *C. speciosa* are summarized in Fig. 3.

### Microstructures of floral organs

**Figure 4:**
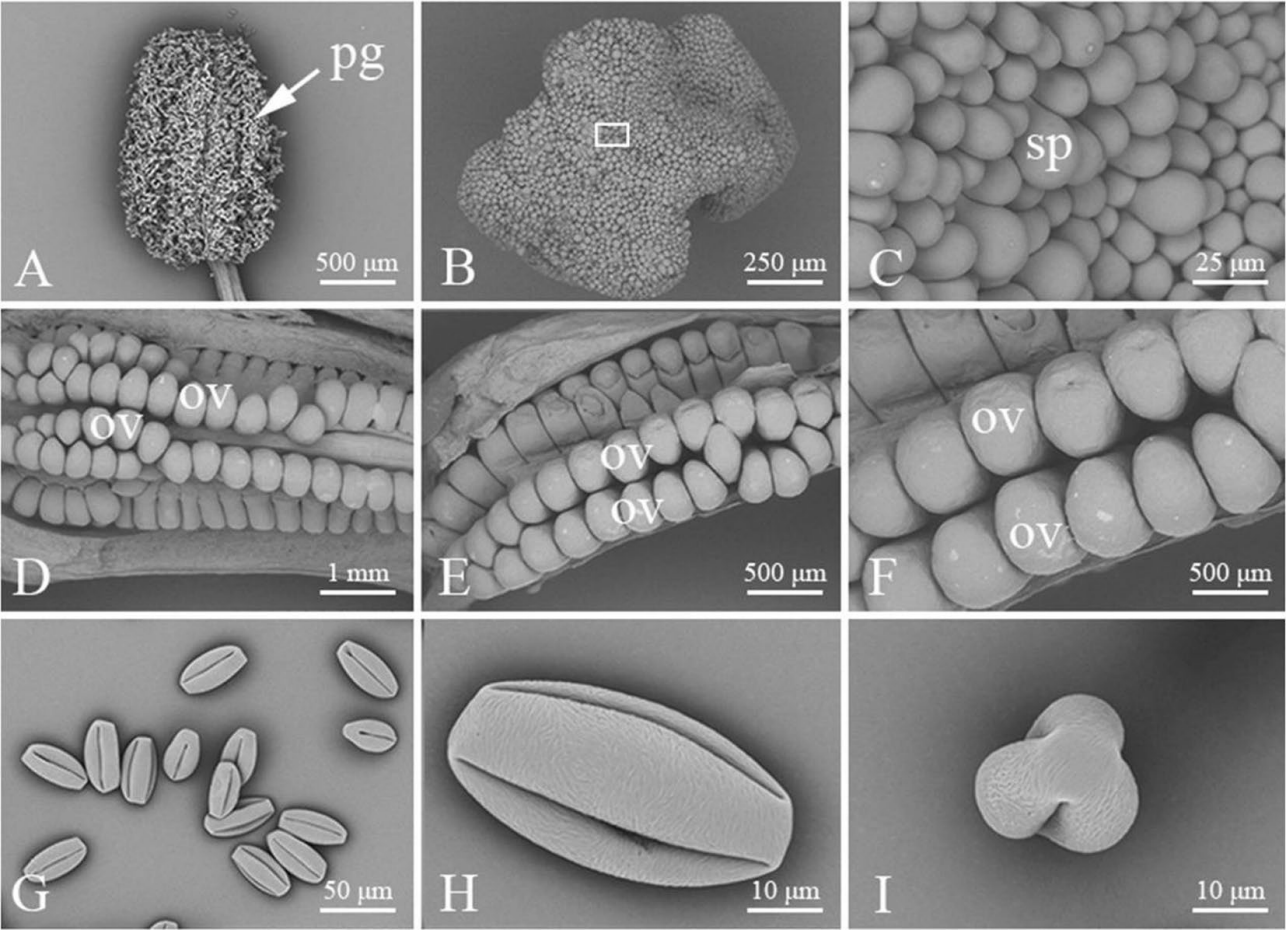
Observations of the floral organs of *Chaenomeles speciosa* using scanning electron microscopy. (**A**) Equatorial view of the pollen-dispersing anther. (**B**) Top view of the stigma. (**C**) Enlarged image of the mastoid cells in the frame in (**B**). (**D**) Irregularly arranged ovules. (**E**) Two-row-arranged ovules. (**F**) Enlarged image of the ovules in the frame in (**E**). (**G**) Clustered pollen. (**H**) Equatorial view of the pollen. (**I**) Polar view of the pollen. Ov: ovule; pg: pollen grain; sp: stigmatic papilla.

**Figure 5:**
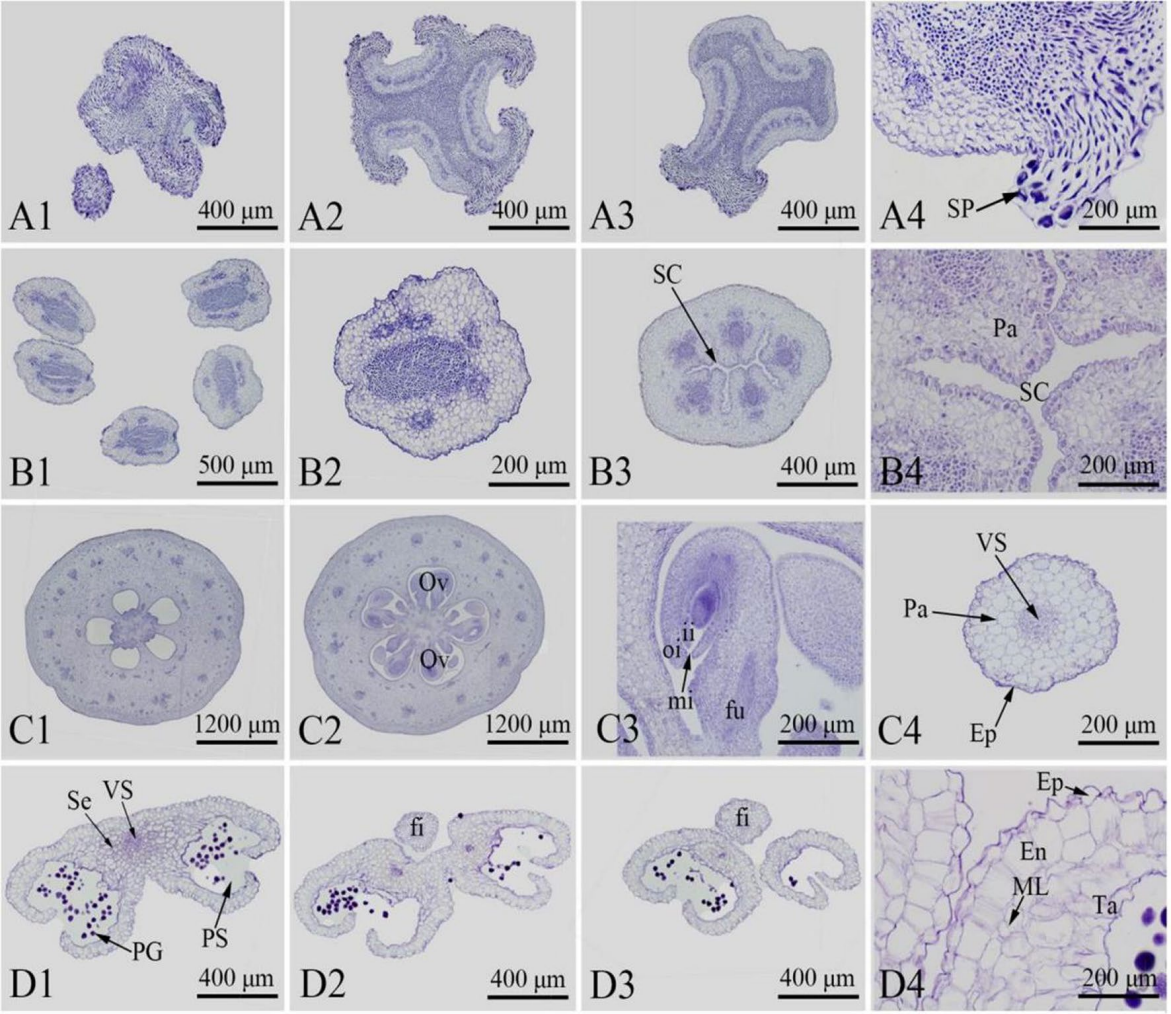
Observations of the transections of the paraffinized sections of the floral organs of *Chaenomeles speciosa* using scanning electron microscopy. (**A1**) Upper part of the stigma. (**A2**) Middle part of the stigma. (**A3**) Lower part of the stigma. (**B1**) Upper part of the style. (**B2**) Lower part of the style. (**B3**) Middle part of the filament. (**C1**) Upper part of the ovary. (**C2**) Middle part of the ovary. (**C3**) Ovule. (**D1**) Upper part of the anther. (**D2**) Middle part of the anther. (**D3**) Lower part of the anther. Ep: epidermis; En: endothecium; fi: filament; fu: funiculus; ii: inner integument; ML: middle layer; mi: micropyle; Ov: ovule; oi: outer integument; Pa: parenchyma; PG: pollen grain; PS: pollen sac; SC: style canal; Se: septum; SP: stigmatic papilla; Ta: tapetum; VS: vascular bundle.

The microstructures of the floral organs were observed under a scanning electron microscope (Fig. 4). The stigma of *C. speciosa* had no secretions and thus was considered the dry type, with many papillae (Fig. 4C). Paraffinized sections were cut, and observations were performed (Fig. 5). The cross section of the paraffined sections showed that the stigma was composed of epidermal cells and basic tissues (Fig. 5A1–A3). There were five styles whose bases were united. The styles had hair and a hollow tubular structure. The paraffined sections showed that the stigma was composed of epidermal cells, basic tissues and intraepidermal cells (Fig. 5B1–B3). The anthers were long and cylindrical in shape and were 1550.00 ~ 1843.33 μm long (average, 1759.54 μm) and 962.48 ~ 1175.33 μm wide (average, 1038.92 μm). The cross section of the anther was butterfly shaped and composed of four pollen sacs separated by septa (Fig. 5D1–D3). The cross section of the mature anther revealed that the anther was composed of epidermis, a fibrous layer, septa and vascular bundles. The ovary was inferior (Fig. 5C1,C2), with a radius of 1840.43 ~ 2142.00 μm (average, 2001.50 μm). It contained five carpels and five compartments that were radially symmetrical. The ovary consisted of locules, ovules and placenta. The ovules were anatropous (Fig. 5C3) and 532.68 ~ 618.36 μm long (average, 2001.50 μm). The ovules were arranged in two rows on the placental axis. The pollen grains were full and elongated in shape (Fig. 4G–I). The polar axis length (P) was 48.12–52.56 μm (average, 49.74 μm), the equatorial axis (E) length was 22.82–26.18 μm (average, 24.13 μm), and the P/E ratio was 1.94–2.05 (average, 2.04). The typically developed pollen had three germination grooves, which were graded or radially symmetrical. The length of the grooves ranged from 40.80 to 45.55 μm (average, 42.93 μm). The outer wall of the pollen was crisped and composed of mesh ridges or irregular holes.

### Pollen stainability

#### Selection of the best method for pollen stainability determination

**Figure 6:**
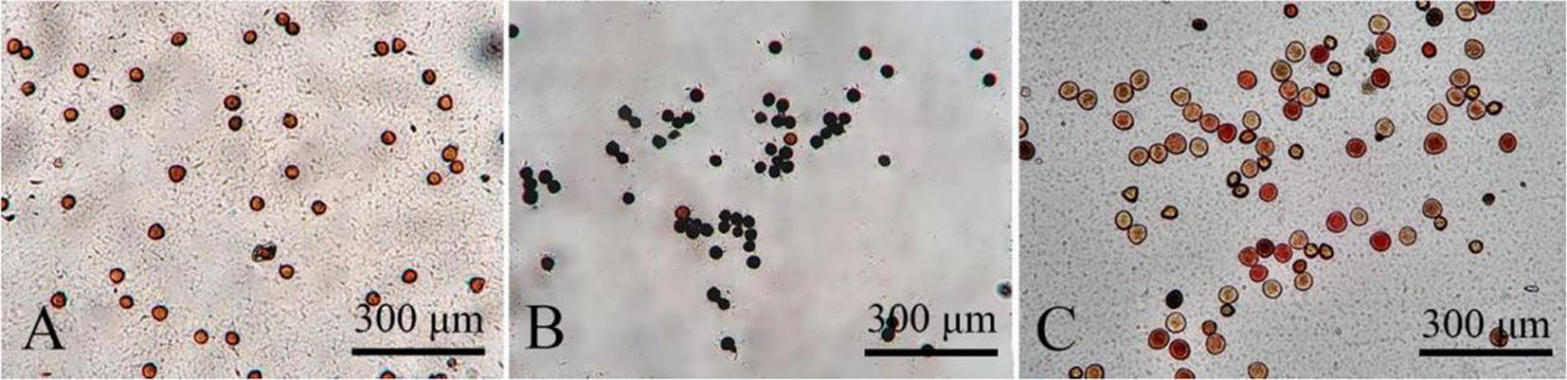
Determination of the pollen stainability of *Chaenomeles speciosa* using different methods. (**A**) Carmine acetate staining. (**B**) I_2_-KI staining. (**C**) TTC staining.

The pollen stainability of *C. speciosa* during the blooming stage was determined separately using carmine acetate staining (Fig. 6A), I_2_-KI staining (Fig. 6B) and TTC staining (Fig. 6C). After carmine acetate staining, nearly all stained pollen grains were red (as high as 98%); therefore, this method was not suitable for the desired use. After I_2_-KI staining, most of the pollen grains were black, with a small number stained red; therefore, this method could not determine the pollen stainability of *C. speciosa*. In contrast, after TTC staining, pollen samples with high viability were stained red, those with low viability were stained light red or pink, and those without viability were stained green or black. Therefore, TTC staining was used to evaluate pollen viability in this study.

#### Pollen stainability in different flowering stages

**Figure 7:**
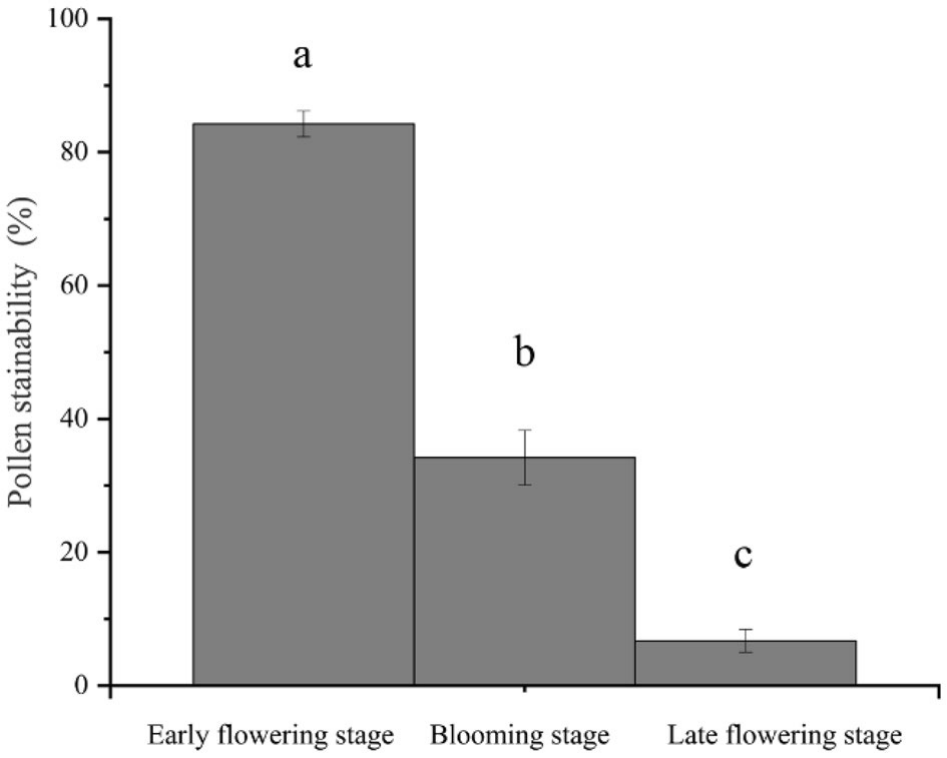
Pollen stainability of *Chaenomeles speciosa* at different flowering stages. Different letters indicate significant differences (*P* < 0.05) according to one-factor analysis of variance.

The TTC staining method was used to determine the pollen viability of *C. speciosa* at different flowering stages (Fig. 7). The pollen stainability of the *C. speciosa* flowers was the highest at the early flowering stage, reaching 84.24 ± 1.96%. The stainability significantly decreased to 34.19 ± 4.09% at the peak flowering stage, which was approximately half of the pollen stainability at the early flowering stage. At the late flowering stage, the pollen stainability was extremely low, reaching 6.72 ± 1.72%. Therefore, *C. speciosa* has the highest pollen viability shortly after the flowers open and the anthers disperse. As the frequency of flowering reaches its peak, a large amount of pollen loses its viability, and ultimately, as the flowering process progresses, the pollen viability gradually decreases.

#### Pollen stainability after different days of flowering

**Figure 8:**
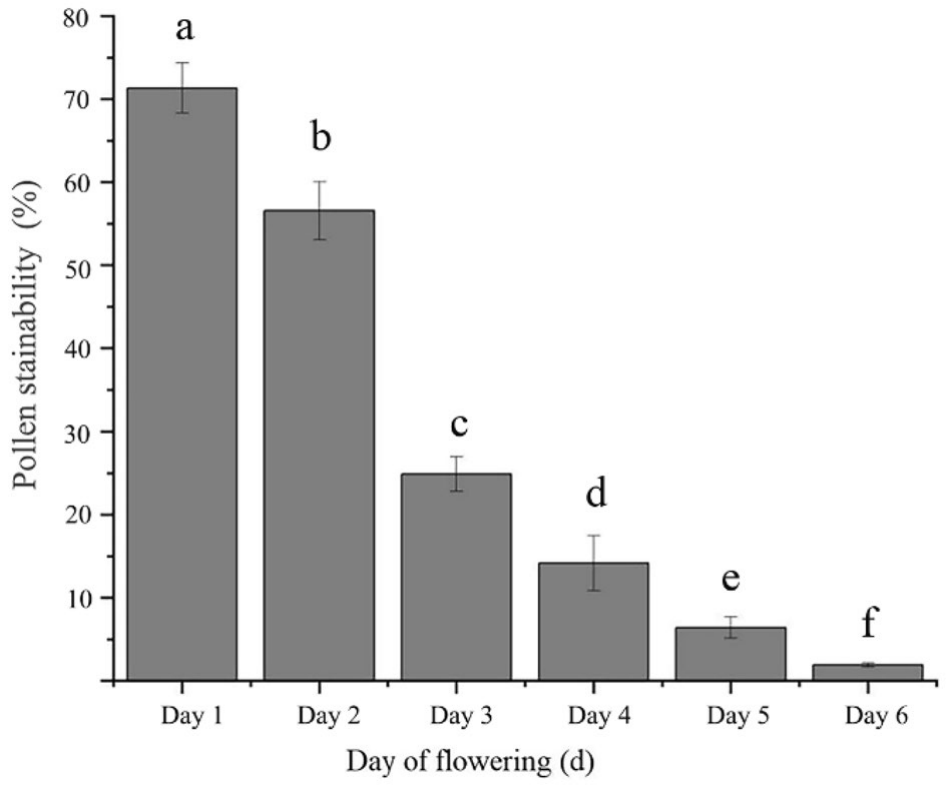
Pollen stainability of *Chaenomeles speciosa* on different days of flowering. Different letters indicate significant differences (*P* < 0.05) according to one-factor analysis of variance.

Pollen was collected on different days of flowering, after which the stainability was determined. According to Fig. 8, the pollen stainability of *C.s speciosa* significantly decreased as the flowers bloomed. On the first day of flowering, the pollen stainability of *C. speciosa* was the highest, reaching 71.75 ± 2.76%. As the flowers grew, the pollen stainability significantly decreased. Two days after flowering, the pollen stainability decreased by 15.19%, and by the third day after flowering, the pollen stainability decreased to 24.49 ± 2.11%. On the sixth day, the stainability dropped to 1.96 ± 0.43%. Therefore, the pollen viability of *C. speciosa* is weak with a short duration.

#### Stainability of pollen stored at different temperatures

**Figure 9:**
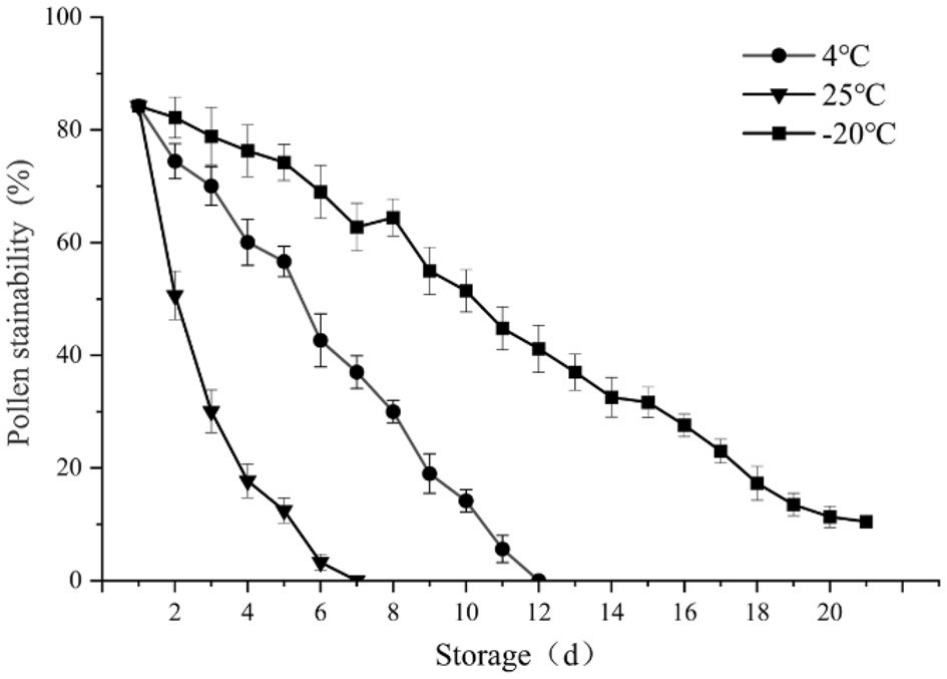
Stainability of pollen of *Chaenomeles speciosa* stored at different temperatures.

The stainability of the pollen stored at room temperature (25 °C), 4 °C and − 20 °C was also determined (Fig. 9), and the results exhibited great differences. Pollen stainability decreased with increasing storage time. The stainability of pollen stored at 4 °C and − 20 °C decreased more slowly than that of pollen stored at room temperature, while the stainability of pollen stored at − 20 °C decreased more slowly than that of pollen stored at 4 °C. The pollen of *C. speciosa* was stored for 12 days at 4 °C, 7 days at 25 °C, or more than 20 days at − 20 °C. Therefore, under normal temperature conditions, the stainability of the pollen decreased very quickly, and the stainability was basically lost within 4 days. These findings indicate that the viability and lifespan of pollen can be appropriately extended by cold storage at – 20 °C.

### Stigma receptivity

**Figure 10:**
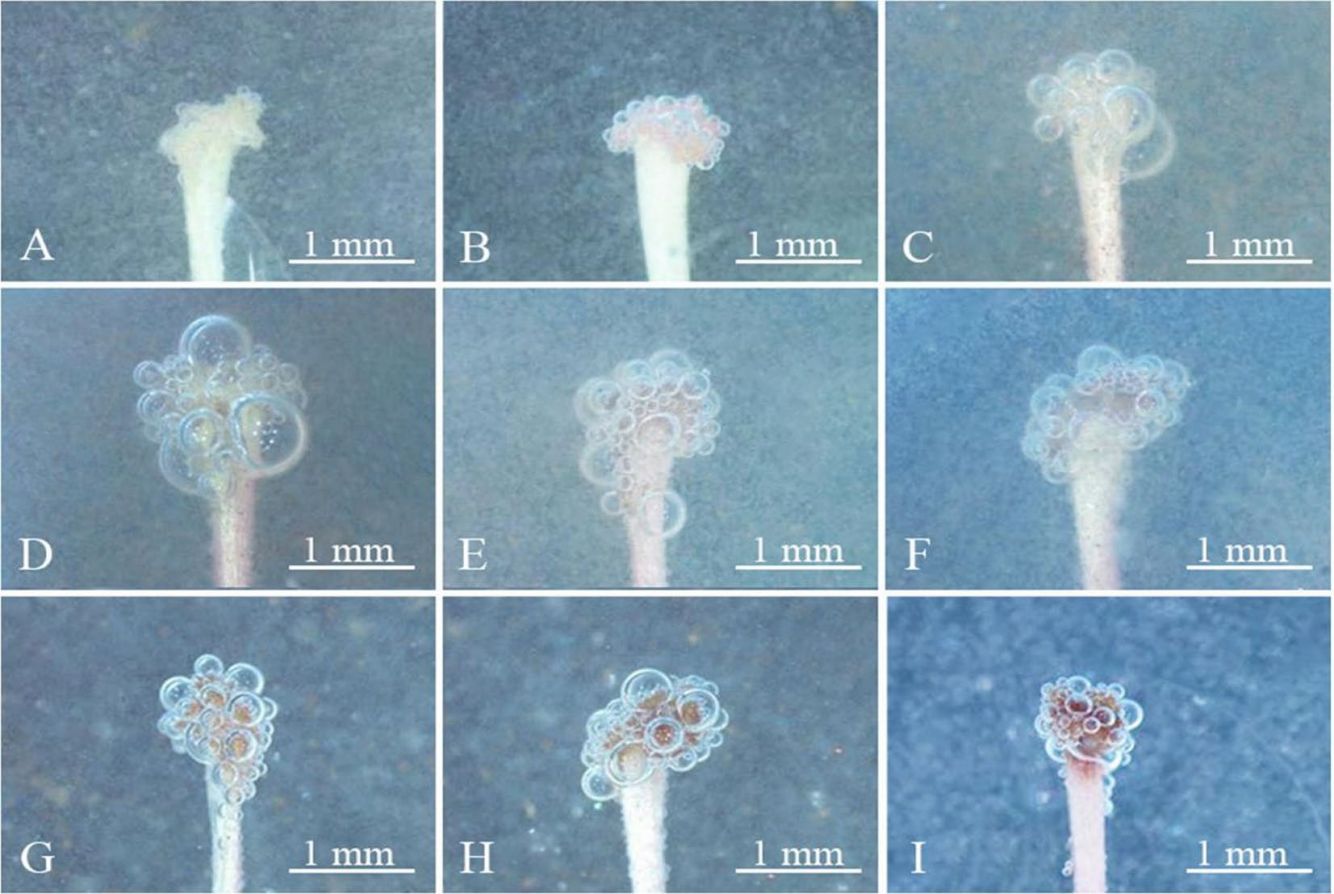
Stigma receptivity of *Chaenomeles peciosa* based on the benzidine-H_2_O_2_ method. (**A**) 2 d before flowering. (**B**) 1 d before flowering. (**C**) On the day of flowering. (**D**) 1 d after flowering. (**E**) 2 d after flowering. (**F**) 3 d after flowering. (**G**) 4 d after flowering. (**H**) 5 d after flowering. (**I**) 6 d after flowering.

Stigma receptivity was determined using the benzidine-H_2_O_2_ method, and the results are shown in Fig. 10. At 2 d (Fig. 10A) and 1 d (Fig. 10B) before flowering, the stigma did not exhibit noticeable reactions, except for a small number of bubbles with a long duration of bubble production. The stigma had poor receptivity. On the day of flowering (Fig. 10C), the receptivity gradually became stronger, and the strongest reaction was observed at 1, 2, and 3 d, when the duration of bubble production was shortest and the amount of bubbles was greatest (Fig. 10D–F). This was the time when receptivity reached the highest level. On Days 4–5 of flowering, the amount of bubbles gradually decreased, and the duration of bubble production began to increase. The stigma began to turn black. On day 6, the stigma became completely black. The number of bubbles was low, and the duration of bubble production increased; moreover, the stigma nearly lost all its receptivity (Fig. 10I). Therefore, the optimal receptivity of the stigma of *C. speciosa* occurs from 1 to 3 d after flowering.

**Figure 11:**
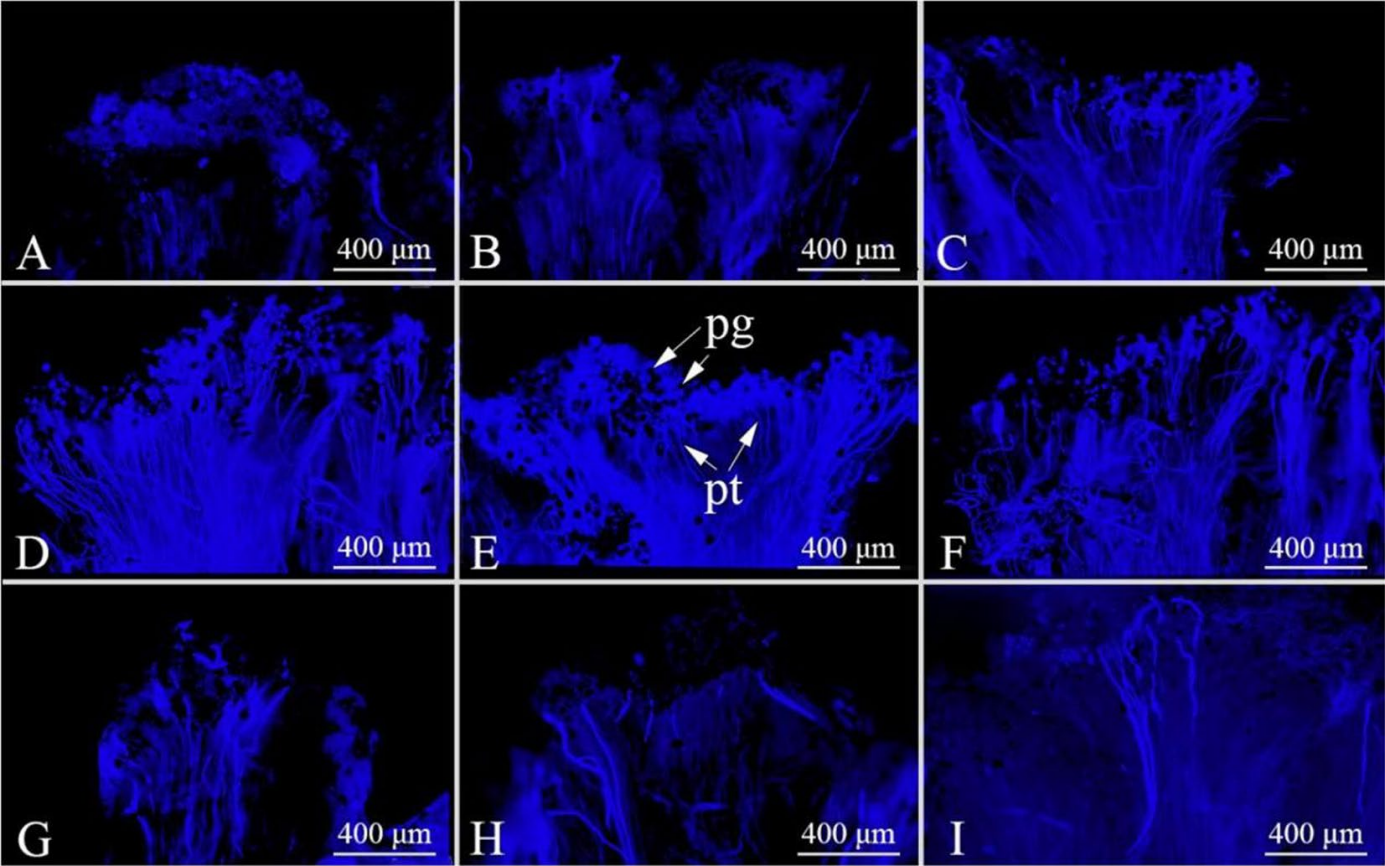
Growth of *Chaenomeles speciosa* pollen after artificial pollination under a fluorescence microscope. (**A**) 2 d before flowering. (**B**) 1 d before flowering. (**C**) On the day of flowering. (**D**) 1 d after flowering. (**E**) 2 d after flowering. (**F**) 3 d after flowering. (**G**) 4 d after flowering. (**H**) 5 d after flowering. (**I**) 6 d after flowering. Pg: pollen grain; pt: pollen tube.

Pollen germination after artificial pollination was observed using a fluorescence microscope (Fig. 11). At 2 d before flowering, a small amount of pollen was attached to the stigma, and no pollen germination was observed (Fig. 11A). One day before flowering, pollen was attached to the stigma, and an extremely small amount of pollen germinated (Fig. 11B). On the day of flowering, the amount of pollen attached to the stigma gradually increased, and the number of germinated pollen grains increased (Fig. 11C). On days 1–3 of flowering, a large amount of pollen attached to the stigma and germinated (Fig. 11D–F). On Day 4, the number of pollen grains attached to the stigma began to decrease (Fig. 11G). On Days 5 and 6 of flowering, the amount of pollen noticeably decreased, with a small portion of germinated pollen remaining (Fig. 11H,I), and the receptivity of the stigma was weak.

The outcomes according to the benzidine-H_2_O_2_ method and according to the observation method were basically consistent.

### Pollen–ovule ratio (P/O)

The P/O was calculated based on 30 flowers using Cruden’s standards. The mean P/O of *C. speciosa* was 3107.52, ranging from 2357.14 to 3107.52 (Table 1). Based on Cruden’s evaluation method, the breeding system of *C. speciosa* falls within the category of obligate xenogamy.

**Table 1:** Pollen‒ovule ratio of *Chaenomeles Speciosa* based on 30 flowers using Cruden’s standards.

Observation index	Minimum (min)	Maximum (max)	Mean	Standard deviation
Pollen amount per flower	492,000.00	612,000.00	534,789.47	53,106.65
Ovule number per flower	150	210	174.21	23.80
Pollen-ovule ratio	2357.14	4020	3107.52	606.84
Breeding system judgment	Obligate xenogamy			

### Outcrossing index (OCI)

The breeding system for the flowers of *C. speciosa* was assessed based on the Dafin outcrossing index (Table 2). The average diameter of the flowers of *C. speciosa* was 44.25 ± 3.94 mm, which was greater than 6 mm; therefore, these flowers were assigned 3 points. When the outer ring of the stamens started pollen dispersion, the stigma had already become receptive, and the pistil matured earlier than the stamen. Therefore, 0 points were assigned. If the stigma was higher than or equally as high as the anther, and thus, 1 point was assigned.

**Table 2:** Dafin outcrossing index of *Chaenomeles speciosa.*

Observation index	Outcome	Observation value
Flower diameter	> 6 mm	3
Time interval between pollen dispersion from anther and stigma receptivity	Protogyny	0
Spatial relationship between stigma and anther	Stigma higher than anther	1
OCI value	4	
Breeding system	Mainly outcrossing, pollinators needed	

### Flower-visiting and pollinating insects

**Figure 12:**
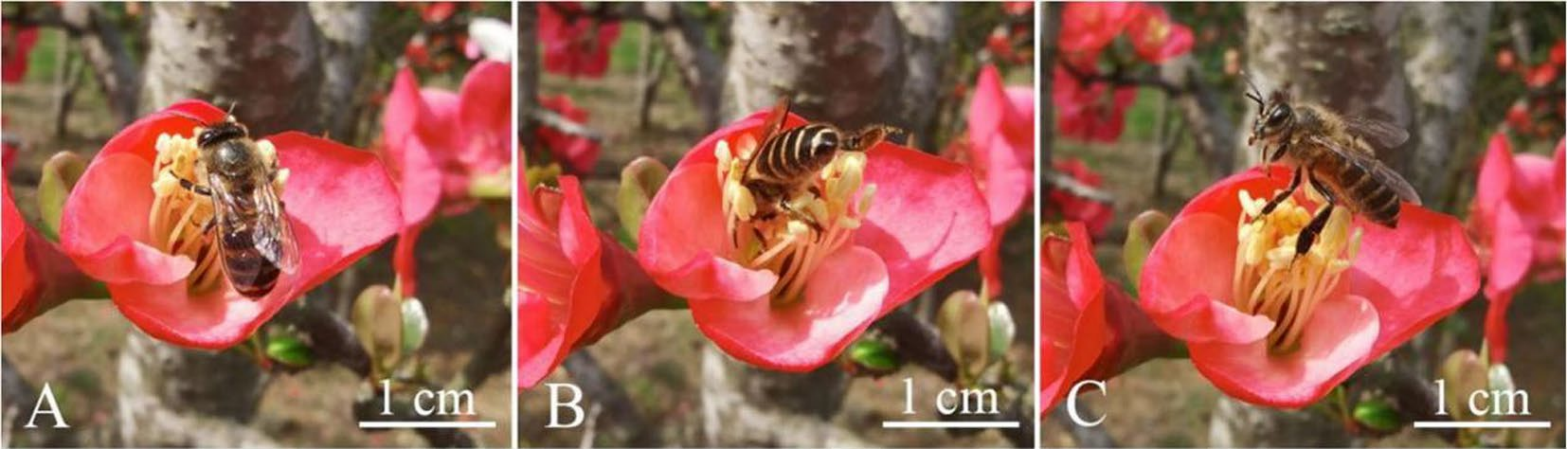
Insects visiting flowers of *Chaenomeles speciosa*. (**A**) *Apis cerana* landed on the stamens. (**B**) *A. cerana* visiting the inside of the pistil cluster. (**C**) *Apis cerana* leaving after pollen collection.

According to the field observations at fixed sites, the temperature was low in the morning and evening in early spring (Fig. 12). As the temperature increased at noon, bee activity occurred, which was particularly frequent during clear weather. During the flowering period of *C. speciosa*, the main visiting insect was *Apis cerana*. The anthers of *C. speciosa* were large and contained a large amount of pollen. When *A. cerana* visited flowers, they penetrated deep into the pistil and stamen clusters. When their back, chest, and legs touched the pollen, they carried a large amount of pollen.

## Discussion

### Characteristics of the flowering biology of C. speciosa

A comprehensive and systematic understanding of the biological characteristics of plant flowering is a prerequisite for studying plant life history and serves as the foundation for studying the protection, utilization, and breeding of germplasm resources^36^. The flowering phenology and typical floral characteristics of plants play important roles in their pollination. The flowering period of *C. speciosa* lasts from March to April, with the flowering period of single plants lasting for 18–20 d. *Chaenomeles speciosa* has a "centralized flowering mode", similar to most plants^37,38^, with single flowers having a flowering period of 8–10 d. A long single flowering period is conducive to pollination and fertilization in adverse environments^39^. *Chaenomeles speciosa* has a flower diameter of 3–4 cm. Flowers with a diameter greater than 2.78 mm are defined as large flowers that can attract visiting insects better and increase pollination opportunities^40,41^.

*Chaenomeles speciosa* has 30–35 stamens and 5 styles, with the base connate. The pistils are slightly taller or as high as the stamens are. This structure can prevent self-pollination to some extent and thus increase hybridization opportunities^42^. The pistil is a unique structure of angiosperms that provides a place for pollen‒pistil interactions and plays a protective role. The diversity of angiosperms depends on pollen‒pistil interactions, such as self-incompatibility and pollen competition^43^. In this study, we observed the floral organs of *C. speciosa* using paraffin sectioning and SEM. The stigma of *C. speciosa* is classified as dry, and its style is hollow. The pollen tube enters the ovary along the style channel. The anthers are cylindrical and butterfly shaped according to the cross section, with 4 pollen sacs. The ovary of *C. speciosa* is inferior, and the inverted ovules are arranged in two rows on the placental axis.

### Characteristics of the breeding system for C. speciosa

The plant breeding system is a key aspect in evolutionary biology research, and there is usually a certain degree of adaptability between the floral characteristics of plants and the plant breeding system^27^. This adaptability is reflected by the morphological characteristics of the flowers, the length of the flowering period, and pollination characteristics, among other aspects. The plant breeding system can be accurately classified based on the P/O value, OCI value, and results of bagging experiments^33^. In this study, the P/O value of *C. speciosa* was approximately 3107.52, which indicates obligate xenogamy. This finding is basically consistent with that reported in the literature^28^. The OCI of *C. speciosa* was 4. This finding indicates that the breeding system of this species involves outcrossing and is partially self-compatible and requires the participation of pollinators. Although the transition from allotypic hybridization to self-pollination is one of the most common changes in plant evolution, only approximately 10–15% of flowering plants are self-pollinated, and outcrossing is the main driving force for plants to exhibit pollination diversity^44^.

Our observations of the pollen stainability of *C. speciosa* in three different flowering stages showed that its pollen stainability was the highest in the early flowering stage, reaching 84.24%. During the single-flower lifespan (approximately 8–10 d), the pollen had high stainability for only a short time, leading to low stamen fitness. Stigma receptivity is an important indicator of flower maturation and affects the transmission rate, self-pollination rate and gametophyte selection at different stages after flowering; furthermore, stigma strength is an important prerequisite for pollination and fertilization^45^. The stigma of *C. speciosa* can accept pollen even before flowering, which is manifested by the observation that the pistil matured before the stamen. This phenomenon, combined with a longer receptive period, can increase the chances of plant outcrossing^46,47^. In this study, the stigma receptivity of *C. speciosa* was measured with benzidine-H_2_O_2_, and only bubbles were observed without a change in stigma color. These findings are similar to those reported for *Aerides rosea*^48^ and *Agastache rugosa*^49^. Based on the bubble generation rate and the size of the bubbles, the stigma receptivity of *C. speciosa* reached the highest level at 1–3 d after flowering and then gradually declined until 6 d after flowering, when the stigma lost all its receptivity. The reasons for these findings may be related to genetic, nutritional, and environmental factors, but the specific reasons remain to be further explored. In addition, in this study, we did not perform a bagging pollination experiment, and determination of the breeding system of *C. speciosa* solely based on the P/O value and OCI value needs to be verified in the future. Furthermore, the preliminary observation in this study indicates that the main pollinator of *C. speciosa* is *A. cerana*, which carries a large amount of pollen and penetrates into the pistil and stamen clusters during flower visits.

### Palynological characteristics of C. speciosa

Pollen grains are produced in the anthers of higher plants. As a sexual reproduction unit and carrier of male genetic material^50^, pollen carries a large amount of genetic information with strong genetic conservation, which is an important basis for research on the origin, evolution, classification and species identification of plants^51^. The morphological characteristics of pollen are controlled by the large amount of genetic information carried by the pollen, and the morphological characteristics of pollen can serve as a basis for species (cultivar) identification. Pollination by insects such as bees is adaptive to the morphological and functional characteristics of pollen^52^. The pollen grains of *C. speciosa* are full and have a long spherical shape, and their outer wall is corrugated, with ornamentation composed of net ridges or irregular cavities. The ornamentation is not clear, as there are few perforations, which is similar to the general morphology of *Malus* pollen.

The vitality of pollen is not only determined by plant genetics but also influenced by the environment. There are significant differences in pollen vitality and quantity among different cultivars^53,54^. Staining and pollen culture are the two frequently used methods for determining pollen viability. The selection of suitable methods for determining the pollen viability of different plants can effectively and accurately determine pollen vitality^55^. Low temperature, drying, and hypoxia are beneficial conditions for pollen storage, and storage methods such as ultralow temperature, vacuum, and rapid freeze-drying are effective methods for pollen preservation^56^.

To date, only a few studies have investigated pollen vitality using *C. speciosa* as the material, and there have been no reports on pollen morphology and stigma receptivity. According to Zhu^57^, TTC staining and methylene blue staining are the most suitable methods for determining the pollen viability of *C. speciosa*. *Chaenomeles speciosa* pollen can be stored for 30 days at 4 °C, 7 days at room temperature, or 50 days at -23 °C. In this study, carmine acetate staining, I_2_-KI staining and TTC staining were separately used to determine the pollen stainability of *C. speciosa,* and the results showed that TTC staining was the most suitable method. According to the results of the present study, the pollen of *C. speciosa* could be stored for 12 days at 4 °C, 7 days at room temperature, or more than 20 days at -20 °C, and these durations are slightly different from those reported in the literature^57^. The reason may be differences in artificial processing or differences in pollen stainability characteristics caused by differences in growth environments, which leads to differences in the experimental results. Therefore, further exploration is needed in the future.

## Conclusions

This study was the first to comprehensively and systematically investigate the flowering biology and breeding system of *C. speciosa*. There is good adaptability between the flowering phenology and floral characteristics of *C. speciosa* and its pollination, and some adaptability also exists between its breeding system and the internal organ development and external morphological characteristics of its flowers. On the first day after flowering, the pollen stainability of *C. speciosa* reached the highest level (up to 84.24%). Low-temperature and dry storage can effectively delay pollen inactivation. The pollen grain structure of *C. speciosa* helps the pollen adhere to the insect surface for long-distance transport and pollination. The stigma had the strongest receptivity at 1–3 d after flowering. Therefore, collecting pollen on the first day of flowering, storing it under low temperature and dry conditions, and then pollinating it on the stigma on Days 1–3 of flowering can improve the successful pollination rate. The breeding system of *C. speciosa* involves outcrossing and partial self-compatibility, and its breeding requires the participation of pollinators. *Apis cerana* may be an effective pollinator of *C. speciosa*. The findings of this study may be of great significance to the cultivation and crossbreeding of *C. speciosa*, and they may also have reference value for the reproductive biology of Rosaceae plants.

## Materials and methods

### Experimental site and materials

The experimental site was located on the campus of Guizhou University, Huaxi District, Guiyang (26°45′ N, 106°66′ E), which has a humid, subtropical monsoon climate. The altitude is 1100 m. There is no severe cold weather in winter and no scorching heat in summer, and the average temperature throughout the year is 14.9 °C, with a frost period of 246 days. The air is highly humid, with an average annual precipitation of 1129.5 mm and an average relative humidity of 77%. The air quality is good. The topography of the original karst basin is dominated by karst mountains, and the habitat is composed of acidic soil alternating with limestone, dolomite, sandstone, and shale, which creates conditions conducive to the formation of various acid-soil plant communities.

In this study, 8-year-old *Chaenomeles speciosa* (Sweet) Nakai, which were found to grow well on acidic soil on the campus of Guizhou University and to have no pests or diseases, were selected as the material. The plants were conventionally pruned and routinely managed with water and fertilizer.

This study was conducted in accordance with the relevant guidelines and legislation of the Guizhou Office of Environmental Protection.

### Methods

#### Opening dynamics of single plants

A total of 30 *C. speciosa* plants of roughly the same size that grew well and had no pests or disease were randomly selected at the budding stage. The plants were labeled and recorded, and their opening dynamics were observed. After most of the flowers entered the budding stage, the buds were observed at 8:30 a.m. every 1–2 days. When the buds were expected to open, the flowering of each plant was observed every hour, followed by observation every week until all the flowers of the same plant withered. Photos were taken, and the dynamic changes in flower opening and withering of each plant were recorded.

#### Opening dynamics of single flowers

A total of 30 buds of roughly the same size that grew well and had no pests or diseases were randomly selected from the 30 labeled plants at the budding stage and then recorded. From the bud burst stage to the time when the floral organs had withered and finally became unviable, the flowering dynamics were observed at 8:30 a.m. each day, and photos were taken. Pollen scattering from the flower, flower color change, flowering duration, petal extension, and dynamic changes in floral organ characteristics were recorded until the flower withered.

#### Floral organ characteristics

At the budding stage, a total of 30 buds exhibiting good growth were randomly selected from the 30 labeled plants. The length and width of the bud and pedicel were measured with a Vernier scale.

At the blooming stage, another 30 flowers that grew well without pests or diseases were randomly selected from the labeled plants. The length and width of the calyx, petals, stamens and pistils and the diameter of the flowers were measured with a Vernier scale, and the number, color and relative positions of the calyxes, petals, stamens and pistils were recorded.

The data were statistically analyzed with Excel 2016.

#### Observation of the microstructures of floral organs

##### Scanning electronic microscopy (SEM)

Fresh pollen, stigmas and styles were collected during the blooming period. After treatment, the specimens were placed in an ion sputtering device (MSP-mini, America), vacuumed and coated for 3–5 min. The specimens were subsequently placed under a scanning electronic microscope (Hitachi TM4000 plus, Japan). Photos were taken, and the length and width of the ovules, pollen and stigmas were measured.

##### Paraffin sectioning

Mature fresh anthers, stigmas, ovaries, and styles were collected and placed in Carnoy's fixative (ethanol:acetic acid = 3:1). After the gas was extracted from the bottle, the samples were placed in a refrigerator at 4 °C for 2 days. The fixative was replaced for another 2-d fixation. The fixative was discarded, and 70% alcohol was added. The samples were then stored again at 4 °C. Hematoxylin staining was performed for 2 d, after which the samples were rinsed for 0.4 d. The concentration of alcohol was varied from low to high for gradient treatment. Then, another treatment with alcohol followed by xylene was performed. The samples were embedded in paraffin at different temperatures. The samples were sliced with a paraffin slicer (Leica RM2235, Germany), which was followed by sticking, drying, dewaxing, sealing, and drying. The sections were observed under a biological microscope (OLYMPUS BX53 (LED), Japan).

#### Pollen characteristics

##### Screening of the optimal method for pollen stainability determination

The pollen stainability of *C. speciosa* was determined separately using carmine acetate staining, I_2_-KI staining and triphenyl tetrazolium chloride (TTC) staining to determine the optimal method. After carmine acetate staining, pollen with high viability turned reddish brown, that with low viability turned pink, and that without viability turned black or green. After I_2_-KI staining, pollen with high viability turned blue, pollen with low viability turned brown, and pollen without viability turned black or green. After TTC staining, pollen with high viability was stained red, pollen with low viability was stained pink, and pollen without viability was stained green. For each treatment, three sections were observed, five fields were selected per section, and for each field, the number of pollen grains should not be less than 30.

TTC staining was the best method for observing the viability of *C. speciosa* pollen.

##### Determination of pollen stainability at different flowering stages

Thirty flowers that grew well and had no pests or diseases were randomly selected at the early, peak and late flowering periods. The pollen of each flower was collected and then mixed well. The stainability was determined using TTC staining. For each treatment, three sections were observed, and five fields were selected per section. In each field, the number of pollen grains was no less than 30.

##### Determination of the stainability of pollen stored at different temperatures

The anthers at the early flowering stage were collected and stored, and the pollen was divided into three portions and stored at 4 °C, 25 °C and − 20 °C. The viability of pollen stored at different temperatures was determined at a set time point every day. For each treatment, three sections were observed, and five fields were selected per section. In each field, the number of pollen grains was no less than 30.

#### Stigma receptivity

##### Stigma receptivity was determined using the benzidine-H_2_O_2_ method

Prior to the experiment, buds about to open were randomly selected and labeled. The buds were then regularly observed. After bud opening, emasculation was immediately performed. The flower was bagged, and its opening date was recorded. Stigmas were collected once per day beginning 1 d before opening until complete loss of receptivity. Three replicates were performed for each collection for observation. The freshly collected stigmas were completely immersed in a benzidine-H_2_O_2_ solution (1% benzidine: 3% H_2_O_2_: water = 4: 11: 22) on a concave glass slide. After 15 min, receptivity was assessed stereoscopically (Leica KL300 LED, Germany) according to the number of bubbles.

##### Observation of pollen germination after artificial pollination

Pollen with high viability was used to artificially pollinate the stigmas that had been emasculated on different flowering days, which were then bagged and labeled. Two days after pollination, the stigma was fixed in Carnoy’s fixative (ethanol:acetic acid = 3:1) for 2 d. The fixative was then carefully removed. Next, 70% ethanol was added, and the stigma was stored in a refrigerator. The stigma was subsequently rehydrated with 50% ethanol, 30% ethanol and ultrapure water. It was softened with 8 mol/L NaOH for 8 h and then kept with ultrapure water overnight. Aniline blue (0.1%) (0.1% aniline blue + 0.15 mol/L dipotassium hydrogen phosphate) was added for 3–4 h of staining^58,59^. The material was routinely pressed and subsequently observed under a fluorescence universal microscope (OLYMPUS BX53 (LED), Japan), after which germination was observed and recorded. Whether the stigma had receptivity was determined based on the presence or absence of pollen attachment and germination on the stigma because receptivity is positively correlated with the number of attached and germinated pollen on the stigma^60^.

#### Estimation of the pollen–ovule ratio (P/O)

The number of pollen grains and ovules were counted, and based on their ratio, the breeding system of *C. speciosa* was assessed^32^. The number of pollen grains was calculated using the method proposed by Yuan^61^. Specifically, 30 buds that had not yet opened were collected from single plants, and 10 anthers from single flowers were placed into a 1.5-ml centrifuge tube. After natural opening and complete pollen dispersion, 1 ml of cellulose enzyme solution was added for 2 min of centrifugation. After the pollen was evenly distributed, 1 μl of the solution was extracted and dripped onto a glass slide. The number of pollen grains was counted under a microscope. Three replicates were performed per treatment. To determine the number of ovules, a total of 30 flowers were randomly selected from the 30 labeled trees. A blade and a dissecting needle were used to cut the ovaries of 30 flowers longitudinally, and the number of ovules on each flower was counted under a dissecting microscope.

The breeding systems were assessed based on the P/O ranges provided by Cruden^29^: 18.1–39.0, obligate autogamy; 31.9–396.0, facultative autogamy; 244.7–2588.0, facultative xenogamy; and 2108.0–195,525.0, obligate xenogamy.

#### Outcrossing index (OCI)

Based on relevant indices, the OCI of *C. speciosa* was calculated^31^, and the breeding system was further assessed. The specific standards are as follows: (1) when the diameter of the flower or inflorescence is < 1 mm, 0 is assigned; when the diameter is 1–2 mm, 1 is assigned; when the diameter is 2–6 mm, 2 is assigned; and when the diameter is > 6 mm, 3 is assigned. (2) If pistils and stamens mature simultaneously or pistils mature earlier during the time interval between anther dehiscence and stigma receptivity, 0 is assigned; if protogyny occurs, 1 is assigned; and (3) when the stigma and anther are at the same height, 0 is assigned; if there is a height difference between them, 1 is assigned. The points obtained from these standards were summed to obtain the OCI value. If the OCI is 0, the breeding system is considered cleistogamous. If the OCI value is 1, the breeding system is obligate autogamy. For values of 2, 3 and 4, the breeding system is facultative autogamy, self-compatibility (nevertheless, pollinators may be needed) and partial self-compatibility (pollinators are needed for outcrossing), respectively.

#### Insects visiting and pollinating flowers

The insects that visited the flowers during the flowering period were observed, and photos were taken. The flower-visiting behavior of the plants was recorded, and insect categories were identified to determine whether the visitor was an effective pollinator.

## Data Availability

The datasets used and/or analyzed during the current study are available from the corresponding author upon reasonable request.

## Abbreviations

P/O: Pollen–ovule ratio
OCI: Outcrossing index
SEM: Scanning electronic microscopy
TTC: Triphenyl tetrazolium chloride

## References

[1] Chu, A. X. Research on the Cultivar Classification of *Ornamental Crabapples* in Henan. Doctoral dissertation (Nanjing Forestry University, 2009).

[2] Qian GZ, Tang GG (2005). Taxonomic Study on the *Malus Miller*. J. Nanjing For. Univ. Nat. Sci. Ed..

[3] Guo L, Cao Y, Quan J, Liu BY (2019). Crabapple in China: Past, present and future. Acta Hort..

[4] Zhou T, Jiang H, Zhang W, Zhang D, Fan J, Zhang Q (2020). 'Zi Dieer' crabapple. Hortscience.

[5] Liu F, Wang M, Wang M (2018). Phenolic compounds and antioxidant activities of flowers, leaves and fruits of five crabapple cultivars (*Malus* Mill. species). Sci. Hortic..

[6] Zeng XQ, Li H, Jiang W, Li Q, Xi Y, Wang X (2022). Phytochemical compositions, health-promoting properties and food applications of crabapples: A review. Food Chem..

[7] Yu CH, Wang M, Liu F, Wang M (2021). Nutrient compositions and functional constituents of 12 crabapple cultivars (*Malus* Mill. species): Aptitudes for fresh consumption and processing. J. Food Process. Pres..

[8] Wang R, Shen X, Zhang W (2018). Analysis of differentially expressed proteins in the pollen of *Malus spectabilis* (crabapples) and *Malus domestica* ('Gala' apples) and their role in pollination. J. Hortic. Sci. Biotech..

[9] Bessho H, Wada M, Kudo K, Inomata Y, Iwanami H, Abe K (2009). Selection of crabapple pollinizers for 'Fuji' and 'Tsugaru' apple. J. Am. Pomol. Soc..

[10] Yin CM, Xiang L, Sun CX, Shen X, Chen XS, Zhou H (2016). Effects of different apple rootstocks on the soil microbial quantity and enzyme activity of apple replanted orchard soil. Acta Horticulturae Sinica..

[11] Zhou T, Jiang H, Zhang D, Fan J, Zhang L, Wang G (2019). ‘Fen Balei’ Crabapple. Hortscience.

[12] Zhang LL, Yin Y, Mao Y, Liu Y, Pang H, Su X (2021). 'Hongyi': A new columnar ornamental crabapple. Hortscience.

[13] Yang T, Li K, Hao S, Zhang J, Song T, Tian J (2018). The use of RNA sequencing and correlation network analysis to study potential regulators of crabapple leaf color transformation. Plant Cell Physiol..

[14] Zhang J, Han Z, Tian J, Zhang X, Song T, Yao Y (2015). The expression level of anthocyanidin synthase determines the anthocyanin content of crabapple (*Malus* sp.) petals. Acta Physiologiae Plantarum..

[15] Zhang SY, Han LY, Zhang H, Xin HL (2014). Chaenomeles speciosa: A review of chemistry and pharmacology. Biomed. Rep..

[16] Wang ZJ, Jin DN, Zhou Y, Sang XY, Zhu YY, He YJ (2021). Bioactivity ingredients of *Chaenomeles speciosa* against microbes: Characterization by LC-MS and activity evaluation. J. Agric. Food Chem..

[17] Sun MJ, Zhao HJ, Liu YC, Ma YN, Tian ZH, Wang HJ (2022). Deciphering the pharmacological mechanisms of chaenomeles fructus against rheumatoid arthritis by integrating network pharmacology and experimental validation. Food Sci. Nutr..

[18] Zhang Y, Xu H, He H, Li X, Feng M, He Y (2020). Total triterpenes from the fruits of *Chaenomeles speciosa (Sweet) Nakai* protects against indomethacin-induced gastric mucosal injury: Involvement of TFF1-mediated EGF/EGFR and apoptotic pathways. J. Pharm. Pharmacol..

[19] Ma Y, Li J, Li J, Yang L, Wu G, Liu S (2022). Comparative Metabolomics study of *Chaenomeles speciosa (Sweet) Nakai* from different geographical regions. Foods.

[20] Yao G, Liu C, Huo H, Liu A, Lv B, Zhang C (2013). Ethanol extract of *Chaenomeles speciosa Nakai* induces apoptosis in cancer cells and suppresses tumor growth in mice. Oncol. Lett..

[21] Zhang L, Cheng YX, Liu AL, Wang HD, Wang YL, Du GH (2010). Antioxidant, anti-inflammatory and anti-influenza properties of components from *Chaenomeles speciosa*. Molecules.

[22] Hu F, Li F, Zheng Z, Sun-Waterhouse D, Wang Z (2022). Surfactant-mediated ultrasonic-assisted extraction and purification of antioxidants from *Chaenomeles speciosa (Sweet) Nakai* for chemical- and cell-based antioxidant capacity evaluation. Molecules.

[23] Huang DD, Jiang SG, Du ZN, Chen YH, Xue D, Wang XJ (2022). Analgesic and anti-arthritic activities of polysaccharides in *Chaenomeles speciosa*. Front Pharmacol.

[24] Xu R, Kuang M, Li N (2023). Phytochemistry and pharmacology of plants in the genus *Chaenomeles*. Arch. Pharmacal. Res..

[25] Bian DB, Zhou JB, Wang Y (2014). Flowering and pollinating specifications of *Chaenomeles speciosa*. J. Anhui Agric. Sci..

[26] Zhang Q, Bao D, Yan C, Yan WW, Liu Z, Hu H (2016). Study on storage characteristics of three species of ornamental crabapple pollen. Shandong Agric. Sci..

[27] Mitchell RJ, Karron JD, Holmquist KG, Bell JM (2004). The influence of Mimulus ringen floraldisplay size on pollinator visitation patterns. Funct. Ecol..

[28] Grant V (1971). Plant Speciation.

[29] Grant V (1981). Plant Speciation.

[30] Barrett SC (2010). Understanding plant reproductive diversity. Phil Trans R Soc Lond B..

[31] Dafni A (1992). Pollination Ecology.

[32] Cruden RW (1977). Pollen-ovule ratios: A conservative indicator of breeding systems in flowering plants. Evolution.

[33] Li T, Liu X, Li Z, Ma H, Wan Y, Liu X (2017). Study on reproductive biology of rhododendron longipedicellatum: A newly discovered and special threatened plant surviving in limestone habitat in southeast yunnan. China. Front Plant..

[34] Hirst, P. M. Advances in understanding flowering and pollination in apple trees. Achieving sustainable cultivation of apples. 109–126 (Burleigh Dodds Science Publishing, 2017).

[35] Christopher DA, Karron JD, Semski WR, Smallwood PA, Trapnell DW, Mitchell RJ (2021). Selfing rates vary with floral display, pollinator visitation and plant density in natural populations of *Mimulus ringens*. J. Evol. Biol..

[36] Shao WH, Diao SF, Dong RX, Jiang JM, Yue HF (2013). Study on geographic variation of morphology and economic character of fruit and seed of *Sapindus mukorossi*. For. Res..

[37] Li ZC, Li J, Lv HY, Cao MH (2013). Flowing phenology features of the rare desert plant *Ammodendron argenteum*. Chin. J. Ecol..

[38] Schlindwein C, Westerkamp C, Carvalho AT, Pinheiro PM (2014). Visual signalling of nectar-offering flowers and specific morphological traits favour robust bee pollinators in the mass-flowering tree *Handroanthus impetiginosus* (Bignoniaceae). Bot. J. Linn. Soc..

[39] Torres-Díaz C, Gomez-Gonzalez S, Stotz GC, Torres-Morales P, Paredes B, Pérez-Millaqueo M (2011). Extremely long-lived stigmas allow extended cross-pollination opportunities in a high Andean plant. PLoS ONE..

[40] Abe T (2006). Threatened pollination systems in native flora of the *Ogasawara (Bonin)*. Islands Ann. Bot..

[41] Eckerter T, Buse J, Foerschler M, Pufal G (2019). Additive positive effects of canopy openness on European bilberry (*Vaccinium myrtillus*) fruit quantity and quality. For. Ecol. Manag..

[42] Opedal ØH (2018). Herkogamy, a principal functional trait of plant reproductive biology. Int. J. Plant Sci..

[43] Williams JH (2012). Pollen tube growth rates and the diversification of flowering plant reproductive cycles. Int. J. Plant Sci..

[44] Wright SI, Kalisz S, Slotte T (2013). Evolutionary consequences of self-fertilization in plants. Proc. R. Soc. B-Biol Sci..

[45] Qiu J, Gao C, Wei H, Wang B, Hu Y, Guo Z (2021). Flowering biology of Rhododendron pulchrum. Horticulturae..

[46] Wada N, Uemura S (2000). Size-dependent flowering behavior and heat production of a sequential hermaphrodite, *Symplocarpus renifolius* (Araceae). Am .J Bot..

[47] Wang XR, Ding GJ (2012). Reproductive biology characteristic of *Jatropha curcas* (Euphorbiaceae). Revista de Biología Tropical..

[48] Wang QY (2021). Pollen vitality and stigma receptivity of *Aerides rosea*. Chin. J. Trop. Crops.

[49] Su YY (2016). Study of the pollen viability and stigma receptivity of *Agastache rugosa* from different areas. Acta Prataculturae Sinica..

[50] Boavida LC, Vieira AM, Becker JD, Feijó JA (2005). Gametophyte interaction and sexual reproduction: How plants make a zygote. Int. J. Dev. Biol..

[51] Bedinger P (1992). The remarkable biology of pollen. Plant Cell..

[52] Zhang HH, Wu H, Zhou Q, Zhao R, Sheng Q, Zhu Z (2021). Flowering characteristics and reproductive biology of *Nymphaea hybrid*, a precious water lily. Sci. Hortic..

[53] Franchi GG, Nepi M, Matthews ML, Pacini E (2006). Anther opening, pollen biology and stigma receptivity in the long blooming species, *Parietaria judaica* L. (Urticaceae). Flora.

[54] Sakata T, Takahashi H, Nishiyama I, Higashitani A (2000). Effects of high temperature on the development of pollen mother cells and microspores in barley *Hordeum vulgare* L. J. Plant Res..

[55] Acar I, Kakani VG (2010). The effects of temperature on in vitro pollen germination and pollen tube growth of *Pistacia *spp. Sci. Hortic..

[56] Ren RF, Li Z, Li B, Xu J, Jiang X, Liu Y (2019). Changes of pollen viability of ornamental plants after long-term preservation in a cryopreservation pollen bank. Cryobiology..

[57] Zhu JR (2016). Studies on measurement methods of pollen viability. J. Hebei For. Sci. Technol..

[58] Burke JJ (2002). Moisture sensitivity of cotton pollen. Agron. J..

[59] Hirasuka S, Zhang SL, Nakagawa E, Kawai Y (2001). Selective inhibition of the growth of incompatible pollen tubes by S-protein in the Japanese pear. Sex. Plant Reprod..

[60] Li YH, Kang XY (2007). Stigma receptivity and its detection methods of white poplars. Acta Botanica Boreali-Occidentalia Sinica..

[61] Yuan DY, Tan XF, Hu QS, Zou F (2008). Study on camellia pollen characteristics and the vitality under different storage conditions. J. Zhejiang For. Sci. Technol..

